# Control of the ductile and brittle behavior of titanium alloys in diamond cutting by applying a magnetic field

**DOI:** 10.1038/s41598-019-40702-7

**Published:** 2019-03-11

**Authors:** W. S. Yip, S. To

**Affiliations:** State Key Laboratory in Ultra-precision Machining Technology, Department of Industrial and Systems Engineering, The Hong Kong Polytechnic University, Hung Hom, Kowloon Hong Kong SAR, China

## Abstract

As a result of extensive investigations into deformation mechanisms of titanium alloys, it has been found that ductile and brittle behavior occurs during diamond cutting of the alloys. Other than implementing ductile regime machining for improving machining performances, in this study, an application of magnetic field in diamond cutting is proposed to enhance the machining performances in both ductile and brittle deformations in diamond cutting of titanium alloys. Results from the experiments showed that under the influence of a magnetic field, the cutting heat at the tool/titanium interface decreased, and surface damages induced from the brittle deformation were remarkably suppressed. The surface quality of both ductile and brittle deformation areas was enhanced in a presence of the magnetic field, which the surface profiles were less distortive with fewer cracks and defects in brittle deformation regions, and the cutting forces at the transition point became less fluctuant and much smoother. This study contributes enhancements of machining performances in ductile and brittle machining in diamond cutting of titanium alloys, increasing the precise level of machined components made with titanium alloys.

## Introduction

Titanium alloys are used extensively in biomedical industries, especially for orthopedic and dental treatments, because of their superior material properties such as excellent chemical resistance, biocompatibility, and fracture resistance^1–4^. Medical components made with titanium alloys are required to be manufactured precisely in order to produce a surface quality suitable for biomedical uses. Although titanium alloys have excellent material properties, they are difficult to cut. Titanium alloys have low thermal conductivity, which causes a localization of high cutting temperature at the cutting zone, deteriorating the tool condition and surface integrity of the machined surface. Also, titanium alloys have high yield strength and their flow stresses dramatically increase when the strain rate is over 10^3^ s^−1^ in some particular deformation conditions^5–7^. Therefore, the deformation regime and mechanism of titanium alloys in machining are reported to be ultimately intricate and complicated, which have led to in-depth investigations.

Machining is defined as a process in which a raw material is cut into a designed shape and size by using a manageable material removal process with desired machining conditions. Once a process involves a cut or removal of materials, deformation mechanisms of the material are raised and commonly they include ductile and brittle deformation modes. Therefore, ductile and brittle deformation regimes are important in machining process as they greatly affect the setting of machining parameters such as depth of cut, feedrate, spindle speed, and final machining outcomes. In a machining process, an occurrence of brittle deformation is not desirable because it deteriorates surface finishing, cracks and voids appear in a brittle region of the machined surface^8–11^. Researchers have reported that brittle materials display a response of plastic deformation when machining relatively small depths of cut^12–14^. Therefore, in order to avoid brittle cracks from materializing on the machined surface, it is recommended that materials be cut with a ductile regime machining at the condition of small cutting depth.

Ductile and brittle behavior is one of remarkable machining characteristics in diamond cutting of ultra-precision machining, which regulates material deformation modes and fracture modes. Ductile and brittle behavior happens simultaneously in machining and changes with different machining conditions, which one of their main deformation factors is temperature^15^. Shimada *et al*.^16^ and Xiao *et al*.^17^ conducted molecular dynamic simulations of brittle and ductile ultra-precision machining of silicon. As high cutting temperature and a small diamond tool tip involved, they proposed that a thermal shock existed at the tool/workpiece interface, which would cause crack propagations after critical machining values, leading to a brittle deformation on the machined area. With the linkage of high cutting temperature in ultra-precision machining, the thermal gradient generated in diamond cutting also affects the brittle and ductile deformations of metals. Gumbsch *et al*.^18^ reported that different materials such as glass, refractory metals, steels and semiconductor crystal exhibited ductile and brittle transition at characteristic temperature with a thermal gradient, which crack initiations were happened at the beginning. Then, cracks generated inside a material propagated as brittle cracks with sharp crack front, furthermore, the materials near the crack tips displayed enough plasticity to slow down. The above literature described the significant effect of temperature and temperature gradient on the tool/workpiece interface on the ductile to brittle behavior of materials.

Ductile machining denoted as a feasibility to machine a material mainly by a plastic flow using single point diamond tool, which the machined area is crack free with mirror grade surface; a material displays a transition from ductile to brittle machining regime on the machined surface when the cutting distance increases above the critical cutting thickness or distance^19^. On the other hand, the brittle and ductile behavior of titanium alloys has rarely been investigated. Yip and To^20^ investigated the brittle and ductile deformation mode of titanium alloys in diamond cutting and reported the discovery of clear transition point, brittle and ductile regions on the machined area of titanium alloys in diamond cutting. Also, the ductile deformation region appeared when the cutting distance was below the critical cutting value. Therefore, in order to implement ductile regime machining of titanium alloys, the machining parameters should be set at a relatively small depth of cut and cutting distance which does not go above the critical cutting value. Some researchers applied ductile machining of materials by controlling the cutting distance in machining processes. Blackley and Scattergood^21^ investigated optimum machining parameters for ductile machining and found that the ductile regime material removal process was highly related to depth of cut, which meant that ductile machining could be achieved by setting a relatively small cutting thickness. Zhang *et al*.^8^ applied an ultrasonic assisted cutting technique to shorten the cutting distance in diamond cutting, and attained ductile machining by reducing the contact distance in an instantaneous cutting area. Generally, however, machining parameters in mechanical machining processes of industrial fields normally are set as various values for components with complicated shapes, especially depth of cut and cutting distance. This leads to larger values of those machining parameters than the critical values in practical cutting practices. Therefore, ductile machining sometime is not workable for practical applications. For this reason, apart from conducting machining processes with limited depth of cut and cutting distance, another adaptable approach is to suppress or minimize the machining characteristics of brittle deformation in machining in order to lower its negative effects on the machined surface. Another machining strategy is to enhance the smooth cutting area in ductile deformation thereby uplift machined surface integrity at both the ductile and brittle deformation regions.

In this study, single point diamond cutting assisted by a magnetic field is proposed to enhance the machining performance of both ductile and brittle deformation in diamond cutting of titanium alloys. In experiments, a magnetic field is superimposed into the diamond cutting process to show the effectiveness of the magnetic influences on ductile and brittle deformation behavior in diamond cutting of titanium alloys.

## Thermal conductivity of titanium alloys/tool interface under a magnetic field

Titanium alloys actually contain magnetic elements reacting to a magnetic field positively, which the magnetic elements include titanium and iron, the magnetic particles discussed in this study would refer to these two elements. According to the material source book^22^, titanium alloys are paramagnetic materials, they show a positive reaction toward a magnetic field as long as a magnetic field is present, meaning the magnetic particles inside titanium alloys are able to align under the influence of a magnetic field and form aggreged structures with highly conductive properties.

Researchers have reported that magnetic particles inside magnetic materials would be aggerated and aligned with the direction of an external magnetic field. This phenomenon is clarified by magnetic filler interactions of magnetic particles in a presence of a magnetic field^23^. In the condition of zero magnetic field intensity, magnetic particles may move randomly to other particles because of the van der Waals forces. In a presence of magnetic field, magnetic dipolar energy is large enough to overcome the thermal energy so that magnetic particles tend to align with the direction of the external field. After that, the aligned particles become linear chains, which are highly conductive paths for transferring heat^24,25^ and therefore the thermal conductivity of magnetic materials are enhanced in a presence of magnetic field. Previous research also identified an enhancement of thermal conductivity of materials under the influence of a magnetic field. Vuppu *et al*.^26^ applied an unidirectional magnetic field using permeant magnets, and discovered that paramagnetic particles were aggregated and formed as a chain structure. Chains initially grew from the static condition, and the length of chains decreased as drag forces increased. Boudenne *et al*.^27^ fabricated a thermally conductive silicone rubber filling with nickel particles under a magnetic field and discovered that nickel particles were aligned inside a polymeric matrix. They confirmed that the thermal conductivity of polyermeric matrix prepared within a magnetic field increased about 100% in comparison to the same sample with a random distribution of particles. Using the same logic in this study, in increase in the thermal conductivity induced from highly conductive magnetic chains under the influence of a magnetic field could be applied into diamond cutting of titanium alloys. The thermal conductivity of titanium alloys in a cutting process is enhanced under the magnetic field influence for quickly dissipating the cutting heat. The thermal conductivity at the titanium alloys/tool interface was enhanced under the influence of a magnetic field. In response to the improved performance of heat transference, the degree of machining characteristic of brittle deformation decreased at relatively low deformation temperatures, and also, the deformation area by ductile machining is improved because of lowering cutting temperature at the tool/workpiece interface. Therefore, the levels of both brittle and ductile deformations in diamond cutting of titanium alloys are reduced and upgraded, respectively.

## Method

**Figure 1:**
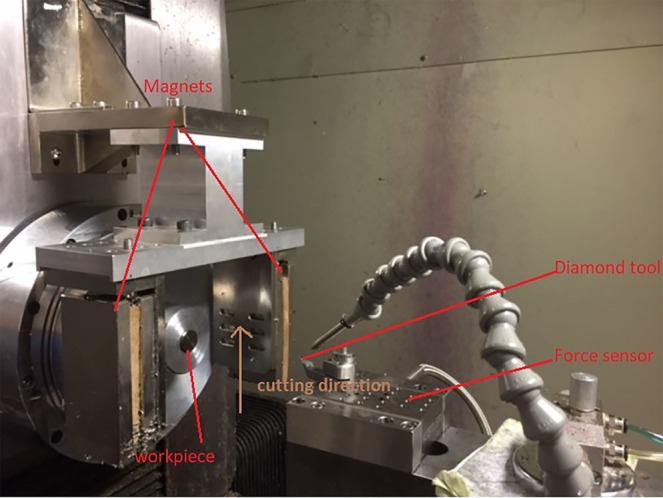
Experimental setup of diamond cutting in a presence of a magnetic field.

Two-phase titanium alloys, Ti6Al4V (TC4), were used as workpiece materials for the experiments. In the experiments, a straight line was cut on the titanium alloy’s surface using a single point diamond tool in a presence of a magnetic field, while a comparative test was conducted by implementing diamond cutting in an absence of a magnetic field. Depth of cut (DOC) was adjusted by five values from 3 µm to 7 µm with 1 µm interval value. Therefore, five straight lines would be cut individually on the machined surface of the two samples. The samples processed the diamond cutting tests in the presence and the absence of a magnetic field, which were named as MFS (magnetic field sample) and NMFS (non-magnetic field sample) respectively. The cutting velocity and tool radius were 150 mm/min and 1.494 mm respectively. A magnetic field was provided by two permanent magnets with a magnetic field intensity of 0.03 T. Titanium alloys were placed at the center of two permanent magnets to expose them to a magnetic field. The experimental setup is shown in Fig. 1. A Moore Nanotech 350FG (4 axis ultra-precision machine) was used as the diamond cutting equipment. Surface roughness, cutting radius and depth of cut of the samples were measured by the Wyko NT8000 Optical Profiling System, which is an optical profiler using non-contact measurement.

## Results

### Machined groove surface and profile

**Figure 2:**
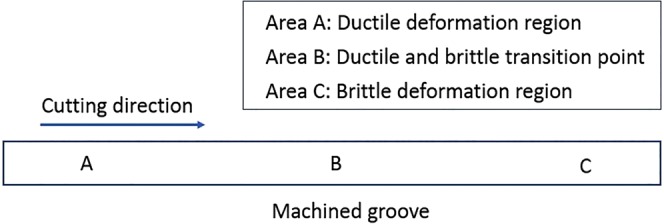
Locations of area A, B, and C on the machined groove of MFSs and NMFSs.

**Figure 3:**
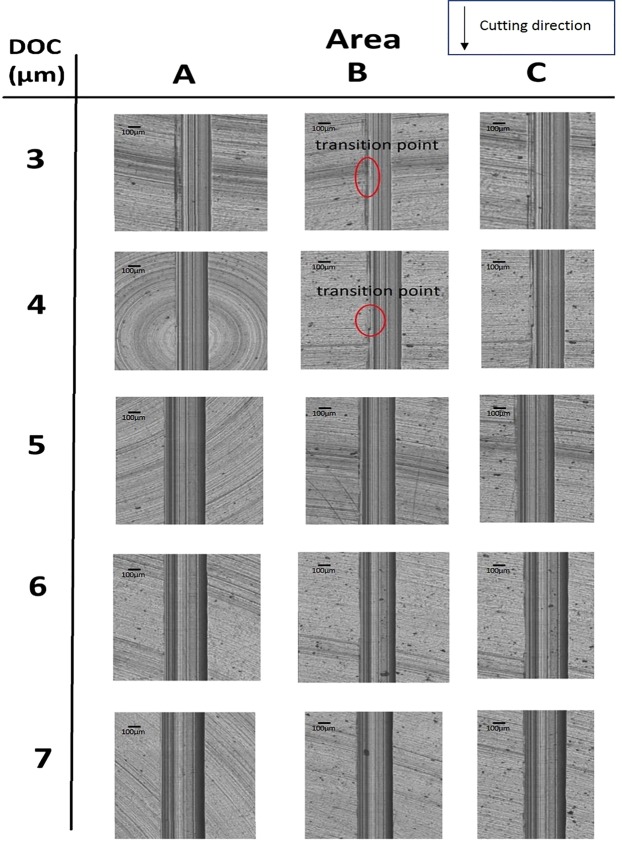
Machined groove surfaces of NMFSs generated at DOC 3–7 μm.

**Figure 4:**
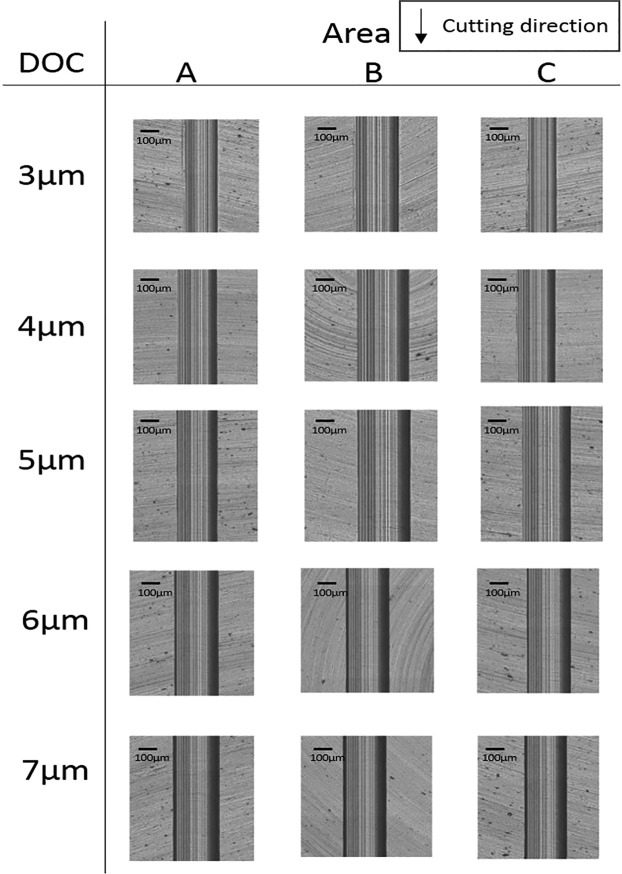
Machined groove surfaces of MFSs generated at DOC 3–7 μm.

In order to evaluate the effectiveness of a magnetic field on suppressing surface damages in a brittle deformation, the machined surfaces and profiles of MFSs and NMFSs were measured. The three areas, A, B, and C, of machined groove were measured and the locations of corresponding areas are shown in Fig. 2. Applications of diamond tool with small size and extremely small DOC cause unique cutting mechanism of ultra-precision machining. In ultra-precision machining of titanium alloys, because of the integral effect of an increase in cutting depth/cutting distance and low thermal conductivity of materials, there exists a high thermal gradient between the cutting area at the diamond tool tip and the surrounding materials near the top surface. This high thermal gradient between small cutting area and the its surrounding materials causes thermal shock. The ductile area at the tool tip and relatively brittle area near the surrounding materials lead an appearance of crack initiation at the cutting area, which starts the transition from a ductile deformation to a brittle deformation. The areas, A, B, and C of NMFSs and MFSs generated under all ranges of DOC are shown in Figs 3 and 4 respectively. According to the results of microscopic views of machined groove at relatively low DOC (3–4 μm), the surface quality of area C was the worst, while the surface quality of area A was the best among all three areas, these proved that ductile deformations were happened at areas A and brittle deformations were happened at areas C. On the other hand, sharp and clear fractural surfaces were displayed at areas B, therefore ductile to brittle transition point was located at area B. For relatively high DOC 5–7 μm, as much higher cutting heat was trapped at the small diamond tool/workpiece interface, brittle fractures were disappeared at all three areas A, B and C. The fully brittle deformation regime was occupied during entire cutting.

In contrast to NMFSs, MFSs displayed superior surface quality at all ranges of DOC. At DOC 3–4 μm, in comparison to NFMSs, smoother groove edges were shown on areas A of MFSs and there was no obvious crack point generated on areas B and C. Especially for DOC 4 μm, a smooth and straight machined groove was generated, with nearly no transition crack at the area B and an extremely low level of uncut surface in the area C. At DOC 5–7 μm, although the fully brittle deformation mode had existed in MFSs too, the machined groove surface and edge of MFSs displayed almost no crack and fissure, which an unswerving and linear groove was formed in the fully brittle deformation regime in diamond cutting of titanium alloys. The above results conclusively verify improvements of machined areas generated by the ductile and brittle deformation regimes under the magnetic field influence, which suppressed the surface damages of brittle deformation and facilitated smooth cutting of ductile deformation in diamond cutting of titanium alloys.

**Figure 5:**
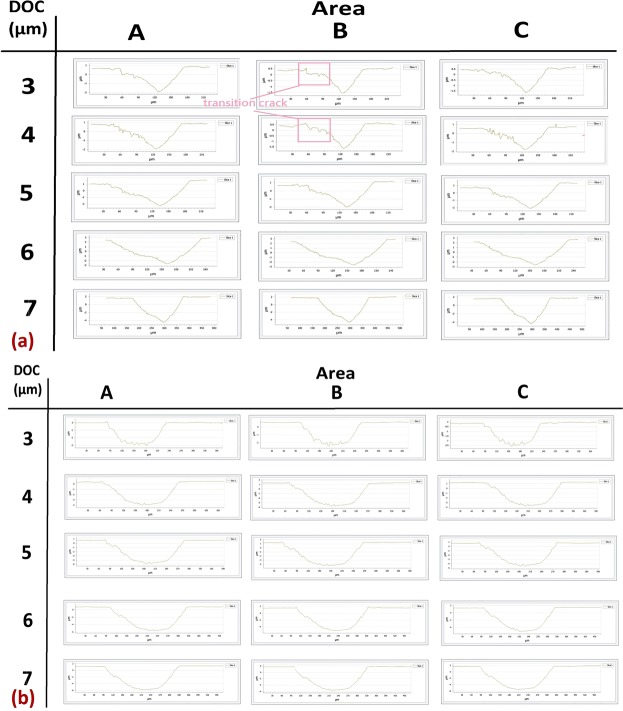
Machined groove profiles of (**a**) NMFSs and (**b**) MFSs generated at DOC 3–7 μm.

The groove profiles of both MFSs and NMFSs are shown in Fig. 5. According to Fig. 5(a), all the groove profiles of NMFSs were distorted to the right-hand side, showing the wavy surface at groove edges especially at the left edges. For DOC 3–4 μm, clear transition cracks displayed at the left sides of the groove edges in area B, which these results were consistent with the results of microscopic of machined surface above and area B was denoted the ductile and brittle transition point. In contrast to NMFSs, for MFSs generated at DOC 3–7 μm as shown in Fig. 5(b), the groove profiles of MFSs showed even shapes with less wavy and fewer cracked surfaces near the edges; the grooves formed as arc curves which were similar to the shape of diamond tool. Even at DOC 3–4 μm, the transition points on the groove crack were blurred and unclear, providing the evidences of the suppression of surface damages in the brittle deformation mode in diamond cutting of titanium alloys.

### Surface roughness and surface quality

Surface roughness of areas A, B, and C of MFSs and NMFSs at DOC 3–7 μm is shown in Table 1. Because of the transition points and brittle facture deformations at areas B and C respectively, surface roughness of these two areas showed higher than that of area A for all samples. On the other hand, surface roughness of areas A, B, and C of NMFSs was much higher than that of MFSs at all range of DOC cutting.

**Table 1:** Surface roughness of NMFSs and MFSs at areas A, B and C.

Depth of Cut(μm)	Average surface Roughness (μm)					
	Area A		Area B		Area C	
	NMFS	MFS	NMFS	MFS	NMFS	MFS
3	181	56	214	63	192	61
4	184	62	221	74	196	70
5	200	76	226	80	216	81
6	206	78	231	82	220	82
7	301	82	312	84	306	89

**Figure 6:**
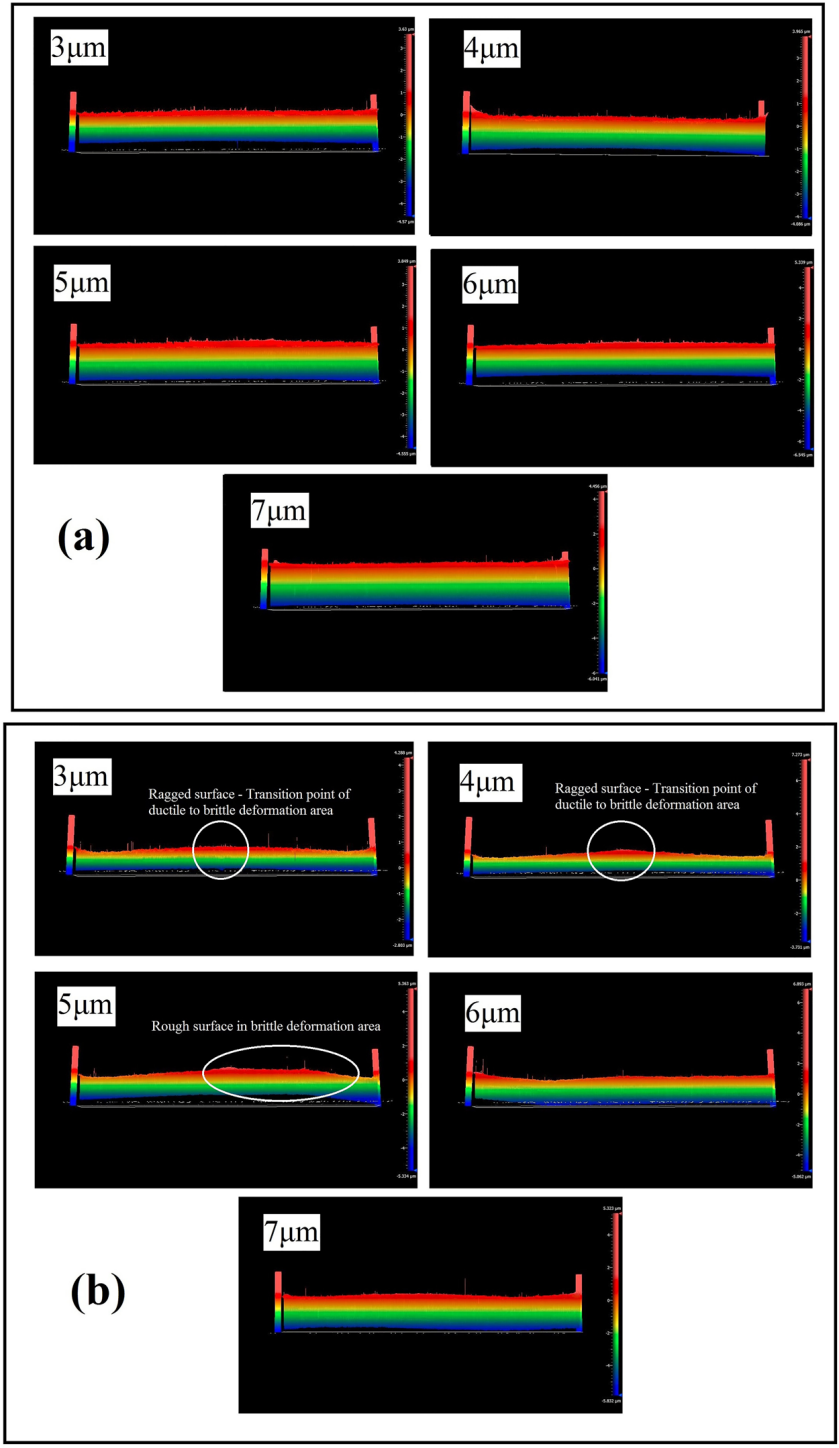
Side view of machined groove of (**a**) MFSs and (**b**) NMFSs generated at DOC 3–7 μm.

The side views of entire machined groove of both MFSs and NMSs were observed under an optical profiler and are shown in Fig. 6(a,b). The ragged surfaces located obviously in the middle part of machined grooves for NMFSs at DOC 3–4 μm, showing the sharp transition points of ductile to brittle area for NMFSs. For DOC 5 μm, the side view of brittle deformation machined area displayed uneven height, which was caused by the brittle fracture in the brittle deformation mode. Conversely, the machined groove sides of MFSs at DOC 3–7 μm showed relatively flat and glossy. No sharp transition point was located at the middle location of the grooves. Also, the machined surfaces with even and stable height were formed at the end part of machined grooves, which were defined as the brittle deformation areas of MFSs. Above information again proves improvements of surface quality and precise level of machined surface generated at both ductile and brittle deformation areas under the influence of magnetic field.

### Cutting force analysis

**Figure 7:**
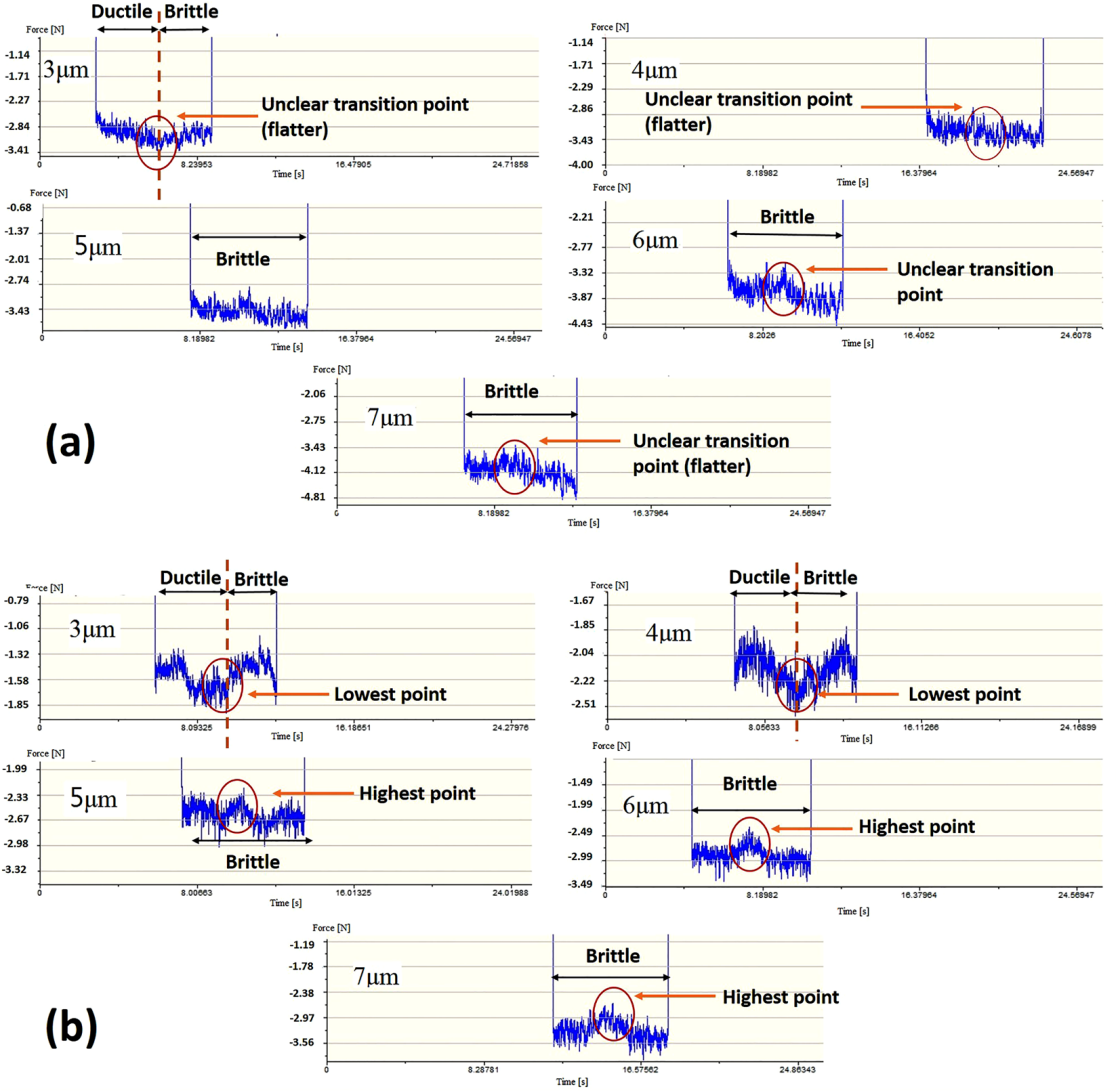
Thrust forces of (**a**) MFSs and (**b**) NMFSs generated at DOC 3–7 μm.

Figure 7(a,b) show thrust forces of both MFSs and NMFSs generated at DOC 3–7 μm. The thrust forces in diamond turning are denoted as signaling an occurrence of material swelling of the machined surface^28–30^, which means that the deformation mode of the machined surface could be reviewed through an observation of the change in thrust forces. For the purpose of observation of change in force trend in diamond cutting tests, therefore the magnitude (y axis) of thrust force can be ignored in this case. According to Fig. 7, the increasing/decreasing patterns of thrust force of MFSs were similar with NMFSs at DOC 3–7 μm. For the thrust forces generated under DOC 3–4 μm, the thrust forces of both MFSs and NMFSs decreased at the beginning and then increased in the following cutting distance, leading to an appearance of the lowest points in between of decreasing and increasing of thrust forces, which was defined as the transition point of ductile to brittle deformation. The changing pattern of decreasing and increasing of thrust forces at a single cut provided an evidence of two different deformation modes when cutting at a constant depth of cut^20^. Similarly, the thrust forces showed a decreasing trend at the beginning region in the experimental results of diamond cutting of titanium alloys, which was the evidence of ductile machining. On the other hand, the brittle fracture deformation generated cracks and fractures on the machined surface consumed higher forces as well as cutting energy. Therefore, the machined surface generated by an increasing thrust force on the deformation area was generated by brittle deformation regime. The ductile and brittle deformations happened at DOC 3–4 μm, while the fully brittle deformation happened at DOC 5–7 μm.

Although the MFSs and NMFSs behaved similar in the deformation modes at all values of DOC, the level and degree of the particular deformation mode (brittle/ductile) were significantly different. For the thrust forces generated at DOC 3–4 μm, the increasing/decreasing slopes of thrust forces generated for MFSs were much gentler than that of NMFSs; the decreasing and increasing slopes for MFSs were smaller than that of NMFSs. Also, the transition points in between the decreasing and increasing forces generated in MFSs were blurred and unclear. Especially for the thrust force at DOC 4 μm, the transition point nearly disappeared and showed as a smooth connection line. On the other hand, as the heat transference under the magnetic field influence increased, the end part of thrust force of MFS at DOC 4 μm was totally dissimilar from that of the NMFS. The thrust force at the end part for the MFS did not increase as same as that of NMFS but rather displayed as a smooth flat line during the remaining part of diamond cutting, while the thrust force of NMF showed a dramatic increase and created a clear transition point. The above implies that the brittle deformation mode in diamond cutting of titanium alloys is relieved and minimized under the influence of magnetic field. The underlying reason was possibility an enhancement of thermal conductivity of titanium alloys in a presence of a magnetic field, which it decreased the thermal gradient between the tool/workpiece interface and the surrounding materials, and therefore the crack initiation was suppressed.

Ductile and brittle deformations happen in diamond cutting of titanium alloys. In some practical situations, diamond cutting processes with parameters over the critical values of cutting depth and cutting distance are unavoidably performed, which they make ductile machining unfeasible. In this study, an application of magnetic field into diamond cutting of titanium alloys was proposed to deliver the feasibility of alternative approach. By employing a magnetic field to diamond cutting of titanium alloys, the surface damages and fractural cracks in the brittle deformation region were minimized, also, the surface quality in the ductile machining region were enhanced.

## Data Availability

The datasets generated during and/or analyzed during this study are available from the corresponding author on reasonable request.
